# Supplementary material for: Knowledge, Attitude, Behavior Practices and Compliance of Workers Exposed to Respirable Dust in a Zambian Copper Mine
Source: Int J Environ Res Public Health. 2023 Sep 20;20(18):6785. doi: 10.3390/ijerph20186785 (PMC10531042; doi:10.3390/ijerph20186785)
Supplement: Supplementary file 1 [file ijerph-20-06785-s001.zip › ijerph-2527388-supplementary.pdf]

Questionnaire for a Study on Mine Worker's Knowledge, Attitudes and Practice Based on the Susceptibility of Health Risks and Dangers of Respirable Dust Exposure.

Thank you for accepting to be part of the study. This questionnaire focuses on Assessing Knowledge, Attitudes, Practice and Compliance related to respirable dust among mine workers.

S.No.	Category	Questions	Response
		1. Site	A. Surface<br>B. Underground
		2. Gender	A. Male<br>B. Female
		3. Age	A. 18 to 25 years<br>B. 26 to 35 years<br>C. 36 to 45 years<br>D. 46 to 55 years<br>E. Above 55 years
		4. Highest education level	A. Primary<br>B. Secondary<br>C. Tertiary
		5. Employment status?	A. Permanent<br>B. Temporary
		6. Job Title?	A. Operator<br>B. Rock Breaker<br>C. Attendant<br>D. Workman<br>E. Person in Charge<br>F. Others
		7. Service years in this Mine?	A. Below 5 years<br>B. 5 to 10 years<br>C. Above 10 years
		8. Total working hours per day	A. 8 Hours<br>B. 12 Hours

Likert Scaled Items Under Variables

For each of the following statements, please indicate by ticking (√), the extent to which you agree with them, using the following scale: (1 = Strongly Disagree; 2 = Disagree, 3 = Neutral, 4 = Agree and 5 = Strongly Agree).

Knowledge

**S/N**	**Items under Knowledge**	**Responses**				
		1	2	3	4	5
1.	I know the type of chemical hazards arising from work in this mine.					
2.	I have knowledge about the health effects that could arise from respirable dust exposure.					
3	Dust exposure can provoke chronic respiratory diseases like silicosis, tuberculosis and lung cancer					
4.	Dust personal protective equipment is relevant to me whilst in our work place					
5.	I know personal protective equipment that can protect one from respirable dust.					
6.	I have knowledge of work place safety rules regarding respirable dust					
7.	The mine/employer have an obligation to maintain employee health and safety.					
8.	I have a source of information about occupational health and safety at the work place					
9.	I know that respirable dust can permanently damage my lungs					

Attitude

**S/N**	**Items under Attitude**	**Responses**				
		1	2	3	4	5
1.	I believe that exposure to respirable dust is hazardous					
2.	As an employee, I believe I should always use dust PPE during work					
3.	I believe that wearing of dust PPE reduces my chances of exposure to respirable dust					
4.	It is important for me to follow safety rules					
5.	I believe the use of dust PPE is relevant to protecting workers					
6.	I believe that safety training in relation to dust exposure is relevant for workers.					

Working Behavior and Practice

S/N	Items under Behavior and Practice	Responses				
		1	2	3	4	5
1.	I obtain dust PPE from within the company on a regular basis					
2.	I use dust PPE during the entire work shift					
3.	I always wear dust PPE during operations					
4.	We are provided with dust PPE at intervals prescribed by the company.					
5.	I follow the laid down procedure to report about lost/worn out PPE.					
6.	I go for medical checkups in relation to dust exposure.					
7.	I usually attend safety training concerning dust exposure					

Compliance with Dust Safety Standards

S/N	Items under Compliance and Safety Standards	Responses				
		1	2	3	4	5
1.	I am clear about my rights and responsibilities in relation to workplace dust exposure and safety					
2.	I always wear dust protective equipment during operations					
3.	I know the necessary precautions I should take concerning dust exposure while doing my job					
4.	Filters in the dust protective equipment are changed regularly					
5.	I know where to report when there is over exposure in my workplace					
6.	At my workplace, systems are in place to identify, prevent, and deal with dust related hazards					
7.	I inspect inhalation and exhalation valves of my dust PPE every time before use					
8.	The company health inspector instructs how to use and where to store dust PPE					

Exposure to Respirable Dust

S/N	Items under Behavior and Practice	Responses				
		1	2	3	4	5
1.	Location of my workplace makes me exposed to dust					
2.	The ventilation system in my workplace is not effective to protect me from dust					
3.	I am exposed to dust during my entire shift					
4.	I am exposed to dust between 1-2 hours of my shift					
5.	Lunch break during the shift reduces exposure to dust in my workplace					
6.	Errors in switching off and on of dust control equipment might expose me to dust					
7.	My job makes me susceptible to dust over exposure					
8.	Dust PPE given to me is not effective to protect me from exposure to dust					

" Thank You for Your Participation!"
